# Electricigens in the anode of microbial fuel cells: pure cultures versus mixed communities

**DOI:** 10.1186/s12934-019-1087-z

**Published:** 2019-02-19

**Authors:** Yujin Cao, Hui Mu, Wei Liu, Rubing Zhang, Jing Guo, Mo Xian, Huizhou Liu

**Affiliations:** 1 CAS Key Laboratory of Biobased Materials, Qingdao Institute of Bioenergy and Bioprocess Technology, Chinese Academy of Sciences, Qingdao, 266101 China; 2 Shandong Key Laboratory of Biomass Gasification Technology, Energy Research Institute, Qilu University of Technology (Shandong Academy of Sciences), Jinan, China

**Keywords:** Microbial fuel cell, Electricigens, Pure cultures, Mixed communities

## Abstract

Microbial fuel cell (MFC) is an environmentally friendly technology for electricity harvesting from a variety of substrates. Microorganisms used as catalysts in the anodic chamber, which are termed as electricigens, play a major role in the operation of MFCs. This review provides an introduction to the currently identified electricigens on their taxonomical groups and electricity producing abilities. The mechanism of electron transfer from electricigens to electrode is highlighted. The performances of pure culture and mixed communities are compared particularly. It has been proved that the electricity generation capacity and the ability to adapt to the complex environment of MFC systems constructed by pure microbial cultures are less than the systems constructed by miscellaneous consortia. However, pure cultures are useful to clarify the electron transfer mechanism at the microbiological level and further reduce the complexity of mixed communities. Future research trends of electricigens in MFCs should be focused on screening, domestication, modification and optimization of multi-strains to improve their electrochemical activities. Although the MFC techniques have been greatly advanced during the past few years, the present state of this technology still requires to be combined with other processes for cost reduction.

## Introduction

The world’s limited supply of fossil fuels and the impact of fossil fuels on climate change require us to develop alternative energy sources. Among the next generation energy sources, microbial fuel cell (MFC) is attracting wide attention due to its intended use to recover energy in the form of electricity. MFCs are fuel cells that convert chemical or solar energy to electrical energy using microorganisms as the catalysts [1]. Unlike other fuel cells, MFCs do not use precious metal catalysts at the anode [2]. Therefore, MFC technology represents a new and promising approach to generate power in an inexpensive way.

The concept of electric current generation by microorganisms has been conceived for more than 100 years and MFC devices for electricity production have been under intense study for about 50 years [3]. MFCs are similar to any other battery or fuel cell, consisting of two electrodes, an anode and a cathode, which are separated by the electrolytes. The difference is that they use organic compounds as substrates to generate electricity. On the anode, microorganisms oxidize organic compounds to release electrons and protons. The electrons produced during oxidation flow to the cathode through external electric circuit to produce current. In the cathode, electron acceptors react with electrons and protons to produce reduced compounds. The reduction of oxygen is the most common cathodic reaction.

Microorganisms that oxidize organic compounds and transfer electrons to the anodes of MFCs are called electricigens. The term ‘electricigen’ is coined to make a clear distinction in the mechanisms of power generation in MFCs. Electricity generated by electricigens is quite different from other microorganisms. In an MFC system, the electricigen used in the anodic chamber is one of the core factors affecting electricity generation performance. Here, we summarize recent development and advances of the electricigens in the anode. The electron transfer mechanism from the electricigens to the anode is discussed in detail. The performances of pure cultures and mixed communities are compared particularly. Future directions of electricigens in MFCs are also proposed.

## Mechanisms for electron transfer from electricigens to electrodes

**Fig. 1:**
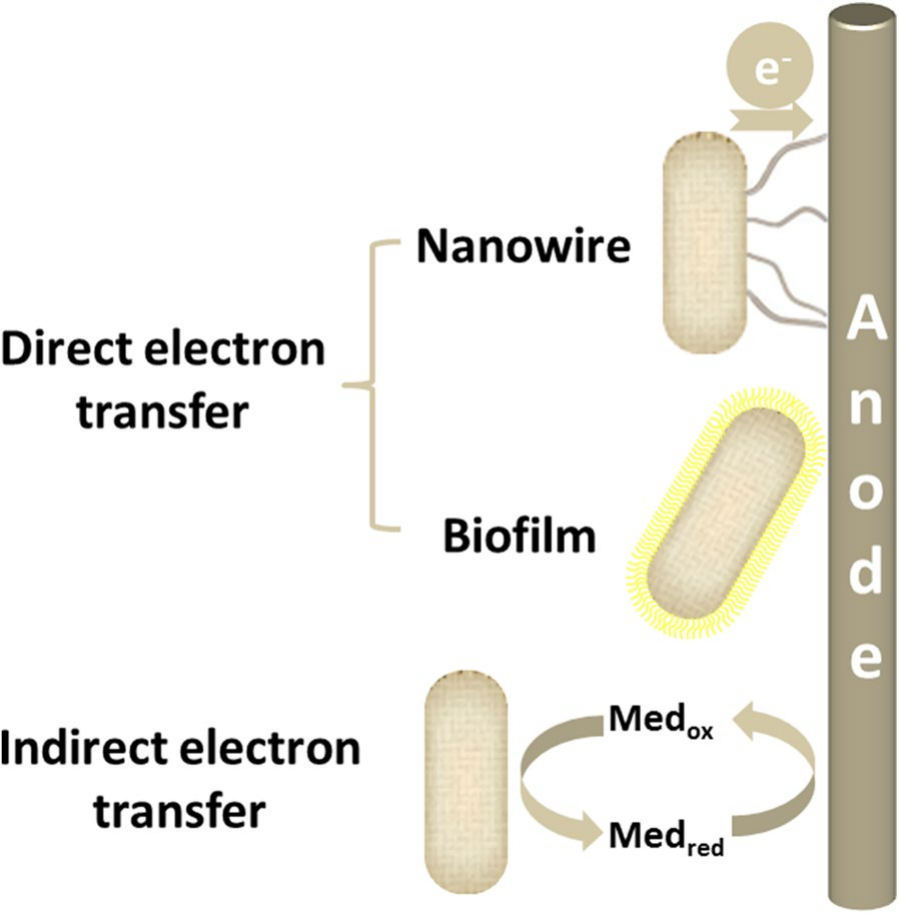
Proposed electron transfer mechanisms from electricigens to the anode. Electrons from electricigens flow to the anode directly through nanowires or indirectly through an electron mediator. Med_red_, reduced electron mediator; Med_ox_, oxidized electron mediator

Electron transfer from the respiration chain of electricigens to the electrode is crucial for MFC technology in harvesting bioenergy. The process of electron transfer by microorganisms is not a natural phenomenon. Although the mechanism is as yet not completely elucidated, multiple pathways for the electron transfer from electricigens to electrodes have been proposed. Generally, these mechanisms can be divided into two types: direct electron transfer (direct contact between the cell surface and the electrode) and indirect electron transfer (through the so-called electron mediators) (Fig. 1).

For direct electron transfer, electrons should reach the outer membrane of the cell and physical contact between the outer membrane and the anode is required. Electricigens form biofilms or electrically conductive nanowires (pili and flagella) on the anode surface [4]. Electron transfer takes place through the outer membrane cytochrome and nanowires or trans-membrane electron transport proteins by direct contact without any diffusional electron mediators. The nanowires are connected to membrane bound cytochromes and allow electricigens to use an electrode which is not in direct cell contact as the electron acceptor. Electron transport proteins play an important role in direct electron transfer as they transfer electrons from the cytoplasm to the outer membrane and finally to the anode. Direct electron transfer is the first choice for efficient current generation in MFCs. The limitation of direct electron transfer is that the active sites of electron transport proteins are typically buried within the proteins, which results in poor electron transfer rate [5]. Up to now, only several species of electrochemically active bacteria such as *Shewanella* and *Geobacter* have been identified to form bacterial nanowires that transfer electrons away from the cell [6, 7].

**Fig. 2:**
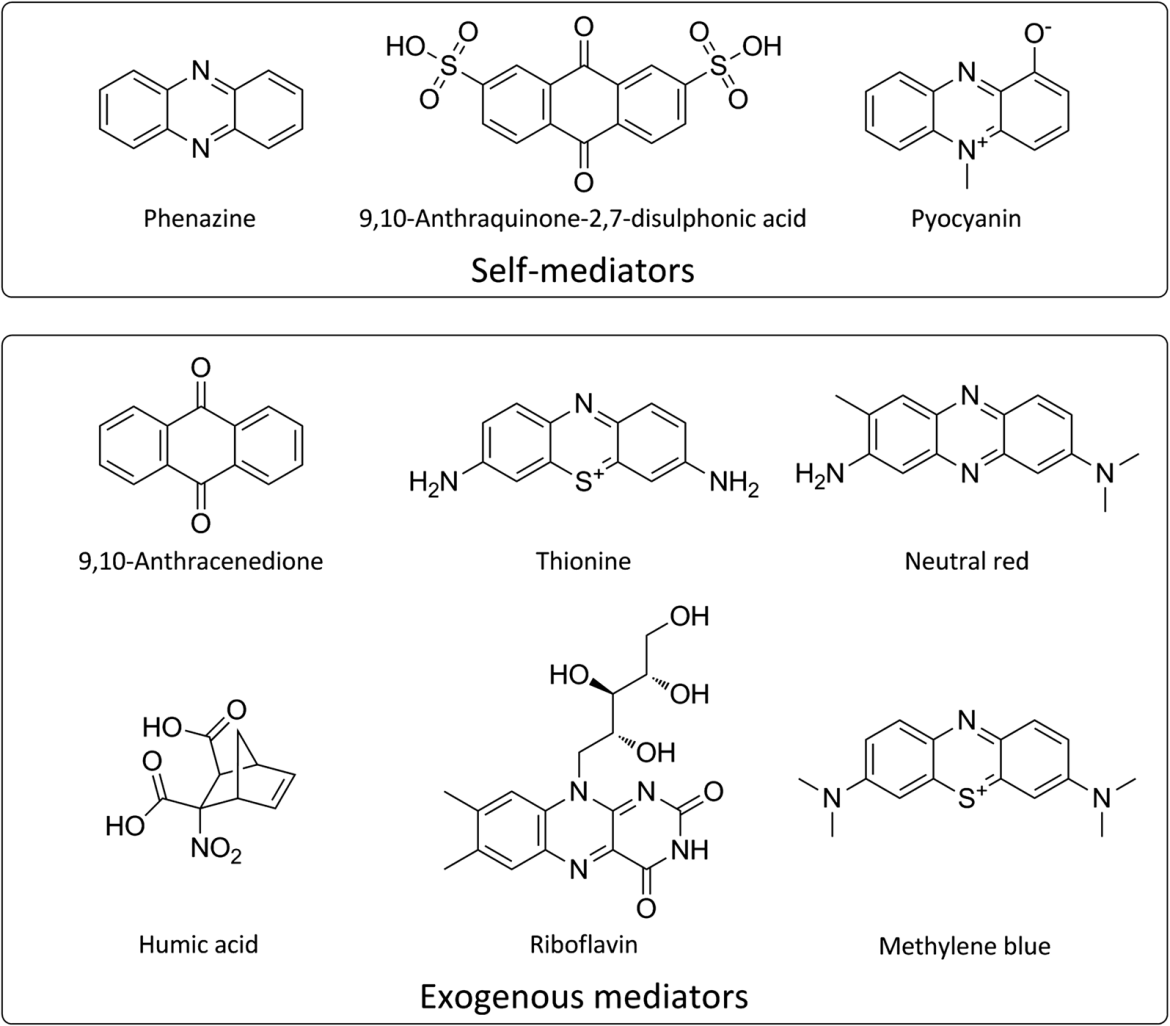
Self-mediators produced by electricigens and exogenous mediators used for indirect electron transfer in MFCs

For indirect electron transfer, electron transfer is achieved with the help of low molecule, soluble mediators (Fig. 2) that eliminate the requirement for direct contact between the cell and electron acceptor. The electron mediators could enter the bacteria cells, extract the electrons from the metabolic reactions of the electricigens and supply these electrons to the anode of an MFC [8]. At first, the presence of electron mediators was considered to be essential for MFC operation [9]. They can be produced by the electricigens or externally added to the anodic chamber. Many species have been identified to synthesize self-mediators such as phenazine [10, 11], pyocyanin [12], and so on. The potential difference between the mediators and the redox proteins would significantly affect the efficiency of electron transfer [13]. A number of chemical compounds like anthracenedione, thionine [14], neutral red [15], humic acid [16], riboflavin [17] and methylene blue [18] have been investigated to improve the efficiency of electron transfer. However, the addition of exogenous mediators is not preferable as they always lead to relatively low current densities as well as being expensive and toxic to the microorganisms, thus causing decline of the performance during long time periods, which makes the technique difficult to commercialize. Moreover, the regular addition of exogenous mediators is technologically unfeasible and environmentally questionable. Hence, if the microorganism can be efficiently used as a catalyst without adding exogenous mediators, it is feasible from a technical point of view that there is no need to gradually add electron mediators as well as being environmentally safe.

## Pure cultured microorganisms as electricigens in the anode

As the biocatalyst of MFCs, electricigens are indispensable. Up to now, hundreds of electricigens have been isolated and used in MFCs. Most of these electricigens belong to Proteobacteria and Firmicutes. Recent studies showed that the electricigens in MFCs had a diverse tendency. Microorganisms that have the characteristics to generate electricity are still waiting to be discovered. In order to further understand the diversity and similarity of electricigens, it is necessary to systematically summarize the existing electricity-producing microorganisms. A summary (Table 1) of the different strains according to the NCBI Taxonomy database that have been isolated from MFCs is given next.

**Table 1:** Overview of MFCs constructed by pure cultures using different electricigens

Type	Genus	Species	Current density	Power density	References
Archaea	*Haloferax*	*H. volcanii*	49.67 μA/cm^2^	11.87 μW/cm^2^	[19]
	*Natrialba*	*N. magadii*	22.03 μA/cm^2^	4.57 μW/cm^2^	[19]
Cyanobacteria	*Synechocystis*	*Synechocystis PCC*-*6803*	NR	72.3 mW/m^2^	[22]
	*Spirulina*	*S. platensis*	NR	6.5 mW/m^2^	[23]
	*Nostoc*	*Nostoc* sp. *ATCC 27893*	2300 mA/m^2^	100 mW/m^2^	[24]
Firmicutes		*C. beijerinckii*	1.3 mA/cm^2^	79.2 mW/m^2^	[27, 28]
Proteobacteria					
α-Proteobacteria	*Rhodospirillum*	*R. rubrum*	NR	1.25 W/m^2^	[32]
	*Rhodobacter*	*R. sphaeroides*	NR	790 mW/m^2^	[33]
	*Rhodopseudomonas*	*R. palustris*	0.99 mA/cm^2^	2720 mW/m^2^	[36]
	*Ochrobactrum*	*O. anthropic*	708 mA/m^2^	89 mW/m^2^	[38]
	*Acidiphilium*	*A. cryptum*	NR	12.7 mW/m^2^	[39]
β-Proteobacteria	*Rhodoferax*	*R. ferrireducens*	31 mA/m^2^	12.9 mW/m^2^	[40, 41]
γ-Proteobacteria	*Escherichia*	*E. coli*	NR	1304 mW/m^2^	[44]
	*Shewanella*	*S. putrefaciens*	NR	1024 mW/m^2^	[47]
	*Shewanella*	*S. oneidensis*	515 mA/m^2^	249 mW/m^2^	[80]
	*Pseudomonas*	*P. aeruginosa*	35 μA/cm^2^	NR	[56]
δ-Proteobacteria	*Geobacter*	*G. sulfurreducens*	7.6 A/m^2^	3.9 W/m^2^	[62]
	*Geobacter*	*G. metallireducens*	125 mA/m^2^	26 mW/m^2^	[65]
	*Geopsychrobacter*	*G. electrodiphilus*	6.6 mA/cm^2^	NR	[66]
Yeast	*Saccharomyces*	*S. cerevisiae*	282.83 mA/m^2^	25.51 mW/m^2^	[70]
	*Candida*	*C. melibiosica*	NR	185 mW/m^3^	[72]
	*Arxula*	*A. adeninivorans*	NR	1.03 W/m^3^	[74]
Eukaryotic algae	*Chlamydomonas*	*C. reinhardtii*	NR	12.95 mW/m^2^	[76]
	*Chlorella*	*C. pyrenoidosa*	NR	6030 mW/m^2^	[77]
	*Chlorella*	*Chlorella* sp. *UMACC 313*	2.83 mA/m^2^	0.124 mW/m^2^	[78]

NR, not reported

### Archaebacteria

Many archaea can survive in extreme environments such as high temperature and salinity which exert tremendous stress to the microorganisms. They have the potential to serve as electricigens in MFCs under special conditions. Two species of halophilic archaea, *Haloferax volcanii* and *Natrialba magadii*, were tested as electricigens in the anode of an MFC. Without any exogenous mediators, the maximum power density and current density reached 11.87/4.57 μW/cm^2^ and 49.67/22.03 μA/cm^2^ for *H. volcanii* and *N. magadii*, respectively. When neutral red was added as the electron mediator, the maximum power density was further improved for both of the archaea and this power output was much higher than *Escherichia coli* under the same conditions [19].

### Acidobacteria

Acidobacteria are physiologically diverse acidophilic bacteria. They can be found in a variety of environments and are able to utilize a wide range of substrates. Several members of this phylum showed electrochemical activity. The iron-reducing bacteria *Geothrix fermentans* was able to produce electron mediators which promoted reduction reaction in the electrode. After optimization of the operation conditions, the current generation rate in the *G. fermentans*-based MFC could reach 0.6 mA and the electron recovery was 97% [20]. Two members of the genus *Arcobacter*, belonging to acidobacteria, were isolated from an acetate-fed MFC. They accounted for about 90% of the population in the MFC which produced a maximum power density of 296 mW/L. These strains specifically associated with the anode of MFCs and negative potentials (− 200 to − 300 mV) were obtained in their pure cultures [21].

### Cyanobacteria

**Fig. 3:**
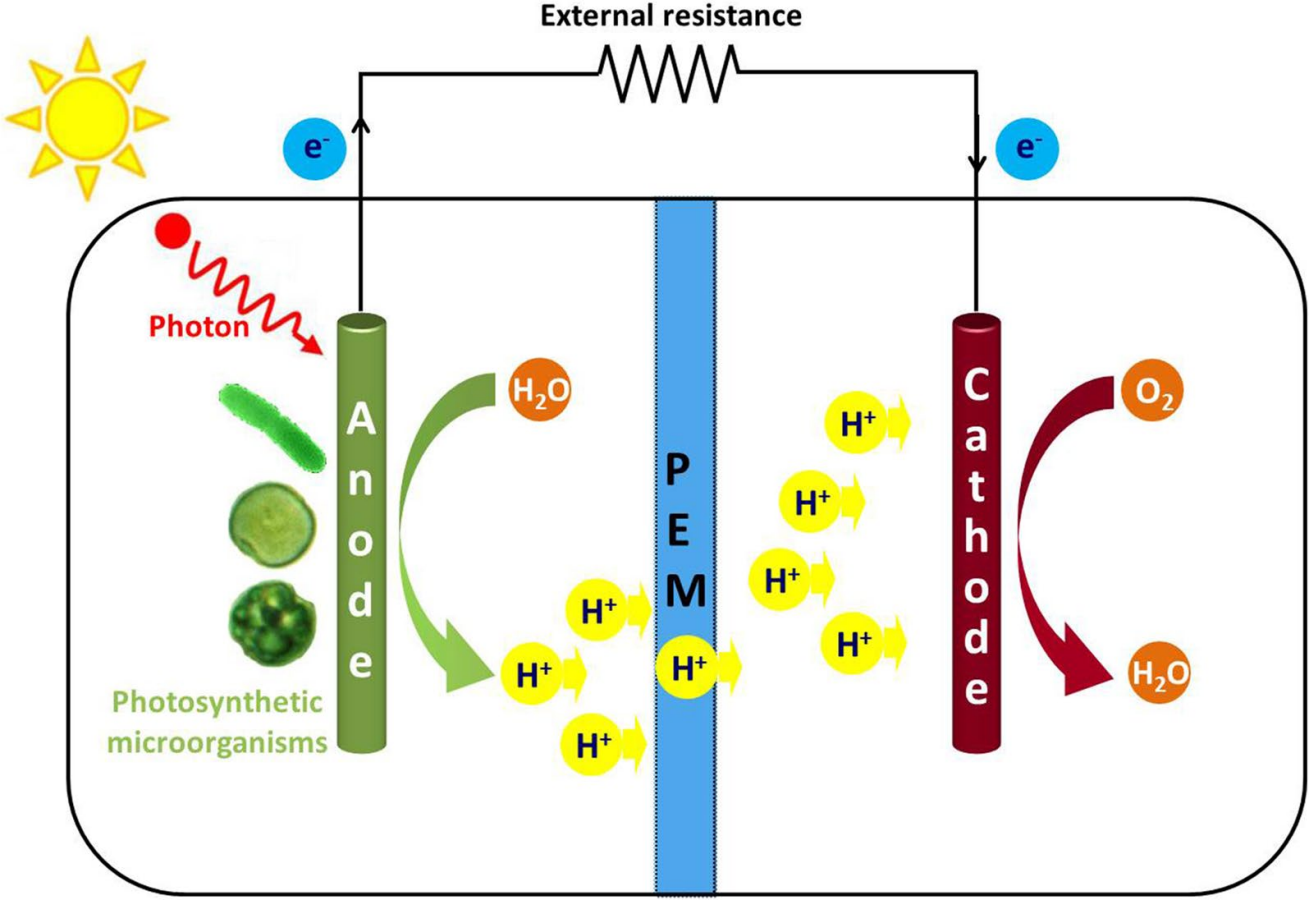
The schematic diagram of a dual chamber PMFC with photosynthetic microorganisms acting as the electricigens in the anodic chamber. PEM, proton exchange membrane

Cyanobacteria are photosynthetic microorganisms and environmentally friendly sources for bioenergy production. During the past few years, many studies have focused on the applications of cyanobacteria in MFCs. The bioelectrochemical systems based on cyanobacteria are called photosynthetic MFCs (PMFCs), which work with light as the power source and generate electricity through the light-driven oxidation of water (Fig. 3). Different species of cyanobacteria have been evaluated as the electricigens in PMFCs. A dual chamber PMFC was constructed using the model cyanobacteria *Synechocystis* PCC-6803. The power output of this PMFC was stable with a maximum power density of 72.3 mW/m^2^ [22]. PMFC using *Spirulina platensis* as the biocatalyst could be operated at high open circuit voltage without externally added feedstocks. The maximum power density obtained by this PMFC reached 6.5 mW/m^2^ [23]. A newly isolated cyanobacterium, *Nostoc* sp. ATCC 27893, was also applied in the anode of a PMFC, which generated current and power densities of 250 mA/m^2^ and 35 mW/m^2^. When 1,4-benzoquinone was added as the electron mediator, a significant improvement in power generation ability was observed (maximum current density of 2300 mA/m^2^ and peak power density of 100 mW/m^2^) [24]. *Synechococcus elongatus* was used as the electricigen to study the response caused by electricity generation. The photosynthetic parameters were determined to clarify the increases of current density. However, electricity generation efficiency of the PMFC was still very low [25].

### Firmicutes

Firmicutes have thick cell walls and are tolerant to harsh conditions. They could be always isolated from mixed cultures in the anode of MFCs. However, electrons need to pass through the cell wall to the anode and thus firmicutes show relatively lower electrochemical activity. *Clostridium butyricum* is a successful isolate of firmicutes which has be applied in MFCs. This strict anaerobe can grow at a wide pH and temperature range. The highest current of 0.22 mA was produced 10 h after inoculation and decreased rapidly when entering the logarithmic growth phase [26]. *C. beijerinckii* from the same genus could also generate electricity with a current density of 1–1.3 mA/cm^2^ from inexpensive substrates, such as starch and molasses [27]. Mutagenesis of *C. beijerinckii* was further performed and the best mutant produced a maximum power density of 79.2 mW/m^2^ in an MFC using glucose as the carbon source and methylviologen as the electron mediator [28]. The firmicute *Thermincola* sp. strain JR was isolated from a thermophilic MFC and demonstrated to be the dominant strain of the electricigens’ community. It generated an average current of 0.42 mA with acetate as the substrate, accounting for approximately 70% of the electricity produced by the entire community [29]. In a methanol-fed MFC, a new strain of firmicutes was isolated and identified to be *Methylomusa anaerophila* by 16S rRNA gene phylogenetic analysis [30].

### Proteobacteria

Proteobacteria represent the largest class of electricigens which are the dominant strains in the microbial communities of MFCs. Many of them have the ability to directly transfer electrons to the electrode. In the proteobacteria phylum, electricigens are widely distributed in *α*-proteobacteria, β-proteobacteria, γ-proteobacteria and δ-proteobacteria.

#### α-Proteobacteria

Several species of *α*-*proteobacteria* are phototrophic bacteria. Therefore, they could also be applied in PMFCs. *Rhodospirillum rubrum* was the first strain used to construct PMFC [31]. The dual chamber PMFC of this bacterium could produce a maximum power density of 1.25 W/m^2^ [32]. Members of the genus *Rhodobacter* are good biocatalysts for PMFCs. Among them, *R. sphaeroides* is the most efficient one. In a single chamber PMFC, the power output reached 790 mW/m^2^ [33]. The current generation ability of *R. sphaeroides* was further enhanced by genetic modification. Two mutant strains, HPC and SDH, were able to produce a current density 50% higher than the wild-type strain [34]. The performance of *R. capsulatus* in PMFCs was also explored but the current density was much lower [35]. *Rhodopseudomonas* can be used for biohydrogen production and they also have the potential to generate electricity. When *R. palustris* was used as the anodic biocatalyst in an MFC, a high power density of 2720 mW/m^2^ was observed [36]. Knockout of the nitrogenase of *R. palustris* further improved its reducing power supply and electricity generation capability. The power density of the mutant was increased from 11.7 μW/cm^−2^ to 18.3 μW/cm^−2^ [37]. Another α-proteobacteria, *Ochrobactrum anthropi*, was isolated from a special U-tube-shaped MFC. The pure culture of this strain could produce 89 mW/m^2^ electricity using acetate as the substrate [38]. Electricity production at relatively low pH was achieved using the acidophilic bacterium, *Acidiphilium cryptum*. The power output reached 12.7 mW/m^2^ with the help of the electron mediators [39].

#### β-Proteobacteria

*Rhodoferax ferrireducens* is the only β-proteobacteria reported as the electricigen in an MFC. It is a facultative anaerobe that can transfer electron to Fe^3+^. Electron mediators were not required in an *R. ferrireducens* MFC system. In a dual chamber MFC, this bacterium produced a current density of 31 mA/m^2^ and the coulombic efficiency reached 81% when using glucose as the substrate [40]. MFC using pure culture of *R. ferrireducens* as the anodic biocatalyst obtained a peak voltage of 0.18 V. The maximum power density reached 12.9 mW/m^2^ in this lab-scale MFC [41].

#### γ-Proteobacteria

γ-Proteobacteria are the most extensively studied electricigens. When Potter et al. demonstrated that electricity could be produced by microorganisms, they used the γ-proteobacteria *E. coli* as the electricigen. *E. coli* is a well-characterized model microorganism and has many advantages e.g., clear genetic background, convenience to be genetically modified and rapid growth property with low nutrients requirements [42]. Genetic tools were employed to engineer *E. coli* to enhance its electricity generation ability. Under anaerobic conditions, the tricarboxyl acid (TCA) cycle of *E. coli* is suppressed, thus leading to low power generation efficiency. Knockout of the *arcA* gene, which encodes an inhibitor of the TCA cycle, greatly improved the performance and power output of the MFC [43]. The endogenous glycerol dehydrogenase was overexpressed in *E. coli* to construct a strain as the anodic biocatalyst. This engineered strain could synthesize electron mediators which promoted electron transfer between *E. coli* cells and the electrode. The peak power density reached 1304 mW/m^2^ in a dual chamber MFC [44]. Disruption of the lactic acid pathway of *E. coli* increased the intracellular reducing power level and electrons production. These electrons were released and then transferred to the anode. A much higher power output was observed when compared to the parental strain [45].

**Fig. 4:**
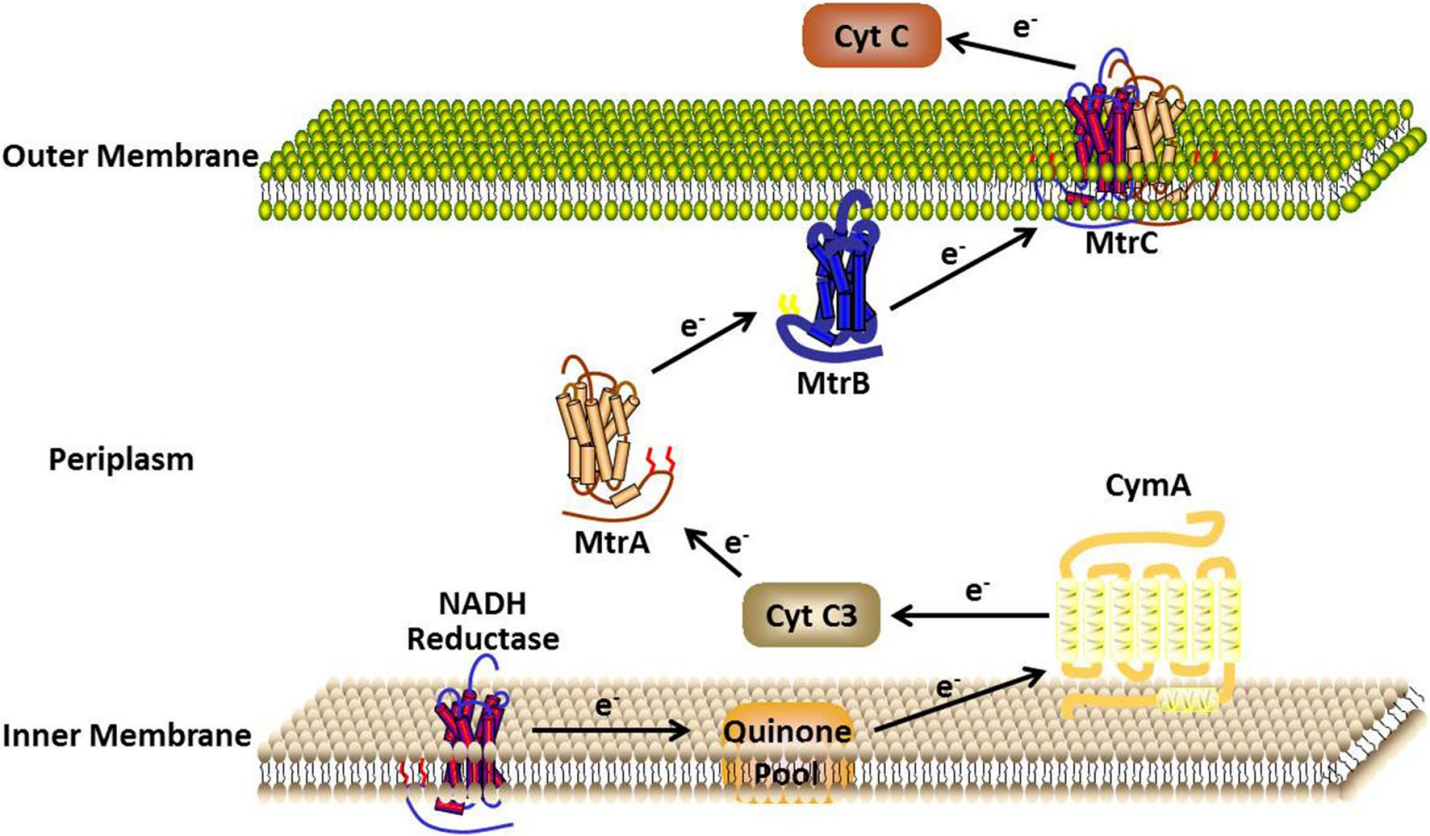
Proposed electron transfer mechanism of *Shewanella*. CymA, inner membrane tetraheme cytochrome; Cyt C3, Cytochrome C3; MtrA, periplasm decaheme cytochrome; MtrB, noncytochrome outer membrane protein; MtrC, outer membrane cytochrome; Cyt C, Cytochrome C

Other important electricigens of γ-proteobacteria are *Shewanella* and *Pseudomonas*. *Shewanella* is widely used in MFCs because of its well-characterized electron transfer mechanisms (Fig. 4). *S. putrefaciens* is the first bacterial strain used to construct a mediator-free MFC. It is a facultative anaerobe capable of reducing metal ions such as iron and manganese. When Kim et al. applied this strain in an MFC, they found it could generate current using lactate as the substrate [46]. A recent study employed uniform nanoflaky nickel oxide array coating strategy to improve adhesion of bacterial cells to the electrode. The performance of the *S. putrefaciens* MFC was improved and a maximum power density of 1024 mW/m^2^ was achieved [47]. The potential of other *Shewanella* strains as electricigens was also evaluated and *S. oneidensis* showed the best capability. Two sub-strains of this species, *S. oneidensis* DSP10 and *S. oneidensis* MR-1, were widely used in different MFCs [48, 49]. A mutant library of *S. oneidensis* MR-1 was constructed using the random transposon-insertion method. One mutant strain, which could generate 90% higher current density, was obtained (insertion of the *uvrY* gene) from the library [50]. A gene responsible for the biosynthesis of cell surface polysaccharide in *S. oneidensis* MR-1 was knocked out. The mutant strain showed enhanced adhesion to the anode and produced 50% more current in an MFC [51]. The *ydeH* gene from *E. coli* was heterologously expressed in *S. oneidensis* MR-1 and the biofilm formation ability of the recombinant strain was significantly improved. The MFCs constructed with this strain produced a peak power density of 167.6  mW/m^2^, about 2.8-fold of the original strain [52]. *Pseudomonas aeruginosa* is the earliest reported strain capable of synthesizing electron mediators. Moreover, *P. aeruginosa* can be genetically manipulated [53]. Biosynthesis of the electron mediators in *P. aeruginosa* was enhanced by engineering the 2-heptyl-3,4-dihydroxyquinoline quorum-sensing system. A maximum current density of 0.5 µA/cm^2^ was achieved by the engineered strain [54]. The expression *PilA* (structure gene coding for protein fibers to form nanowires) from *G. sulfurreducens* in *P. aeruginosa* could yield pili, whose conductivity was comparable with native *G. sulfurreducens* [55]. The *pilT* gene encoding an ATPase could increase the number of pili when it was knocked out. The *pilT* mutant of *P. aeruginosa* was hyperpiliated and reached a peak current density of 35 μA/cm^2^ [56]. The maximum power density was increased by 2.7-fold in compared to the wild-type strain [57].

#### δ-Proteobacteria

δ-Proteobacteria include two important genera *Geobacter* and *Geopsychrobacter*, many species of which could be applied in MFCs. *Geobacter* has the ability to reduce Fe^3+^ using a variety of organic compounds as electron donors [58]. *G. sulfurreducens*, a Gram-negative sulphur-reducing bacterium, produced the highest current among the electricigens isolated up to now. It could attach to the electrode and remain viable for a long period. When this bacterium was first investigated for its electricity generation ability, *G. sulfurreducens* generated electricity from acetate with an electron recovery of 96.8%. The maximum power density of the MFC reached 1143 mA/m^2^ [59]. Then the anode of the MFC was balanced by potentiostat to overcome potential electrochemical limitations and a current density as high as 2.26 A/m^2^ was obtained [60]. Nevin et al. further demonstrated that *G. sulfurreducens* was capable of producing power densities comparable to mixed cultures. The maximum current density and power density reached 4.56 A/m^2^ and 1.88 W/m^2^, respectively. The differences in the power production between mixed communities and pure cultures were attributed to MFC design [61]. In another study, a variant of *G. sulfurreducens* with enhanced capacity for current production was obtained using a simple selection strategy. This mutant strain was much more effective than the wild-type strain. It generated a power output of 3.9 W/m^2^ and a current density of 7.6 A/m^2^ in MFC systems the same as the wild-type strain, representing five- six-fold enhancement of current generation ability [62]. *G. metallireducens* was also an efficient electricigen isolated from MFC devices. It comprised 90% of the anodic communities in sediment-type MFCs operating in rice paddy fields [63]. Power output in an MFC with a pure culture of *G. metallireducens* reached 40 mW/m^2^ [64]. These MFCs could be used for wastewater treatment while generating electricity. The maximum power and current densities were 26 mW/m^2^ and 125 mA/m^2^ when using an MFC inoculated with *G. metallireducens* for domestic wastewater treatment [65]. *Geopsychrobacter electrodiphilus* was an important electricigen isolated from marine sediment MFCs. This bacterium could grow at relatively low temperature and utilize various organic substrates. A peak current density of 6.6 mA/cm^2^ was produced from malate by the *G. electrodiphilus* based MFC with an electron recovery of 85.4% [66].

### Yeast

Various bacteria have been employed as the electricigens. However, there are relatively few studies on eukaryotes as catalysts for MFCs. Yeasts are good candidates as electricigens due to their clear genetic background, fast growth rate and being generally recognized as safe [67]. In Potter’s pioneering research, *Saccharomyces cerevisiae* was also tested for electricity generation [68]. Although yeast MFCs still produce a lower power output than bacterial MFCs, they have received renewed attention. An engineered strain of *S. cerevisiae* with excellent electrochemical activity was constructed by displaying glucose oxidase on its cell surface. The MFC showed higher power output and current density than unmodified yeast [69]. In a recent study, *S. cerevisiae* was used as the electricigen to evaluate electricity production and degradation of substrates under different redox conditions. Using graphite as the anode, higher current and power densities were achieved in a single chamber MFC in the absence of exogenous mediators [70]. Yeast extract was successfully applied in an *S. cerevisiae* MFC as the electron mediator. The addition of yeast extract can enhance the adhesion of yeast cells to the electrode. The maximum current density and power density in this dual chamber MFC reached 300 mA/cm^2^ and 70 mW/cm^2^, respectively [71].

Another yeast strain, *Candida melibiosica*, was also applied as the biocatalyst for MFCs. *C. melibiosica* has high phytase activity and can produce electricity without any exogenous electron mediators, which proved that this strain was an electricigen [72]. Immobilized cells of the yeast *Hansenula anomala* was also tested for its ability to act as the electricigen in a mediator-free MFC and efficient current generation was observed in this system. The presence of redox proteins in cell membranes was thought to contribute to direct electron transfer in the MFC [73]. The non-conventional yeast *Arxula adeninivorans* was another choice as an MFC catalyst. It could transfer electrons to the anode through the secretion of reducing molecules. The maximum power density in the *A. adeninivorans* MFC reached 1.03 W/m^2^, which was one of the most effective yeast MFCs [74].

### Eukaryotic algae

Algae biomass always serves as the substrates for electricigens in MFCs [75]. Moreover, algae can be used as both electron donors in the anode and acceptors in the cathode. In most cases, algae are placed at the cathode of MFCs because they can utilize CO_2_ to generate O_2_ and facilitate the cathodic reaction. Up to now, only *Chlamydomonas reinhardtii* and *Chlorella* sp. have been tested as the electricigens in the anodic chamber. The model microalga, *C. reinhardtii*, was investigated in PMFCs by comparing different light intensities. Red LED light allowed the PMFC to produce a higher power density (12.95 mW/m^2^) than blue light. The higher the light intensity, the better the performance of the PMFC [76]. The green alga *Chlorella pyrenoidosa* was also introduced into the anode of a PMFC. By controlling the culture conditions, this alga could generate electricity without externally added substrates. The maximum power density was relatively as high as 6030 mW/m^2^ [77]. A newly isolated *Chlorella* sp. UMACC 313 was used to form biofilms on the anode. The maximum power and current density in the PMFC reached 0.124 mW/m^2^ and 2.83 mA/m^2^ [78]. The algae were further immobilized in alginate gel within an MFC and the peak power output was enhanced to 0.289 mW/m^2^ [79].

**Fig. 5:**
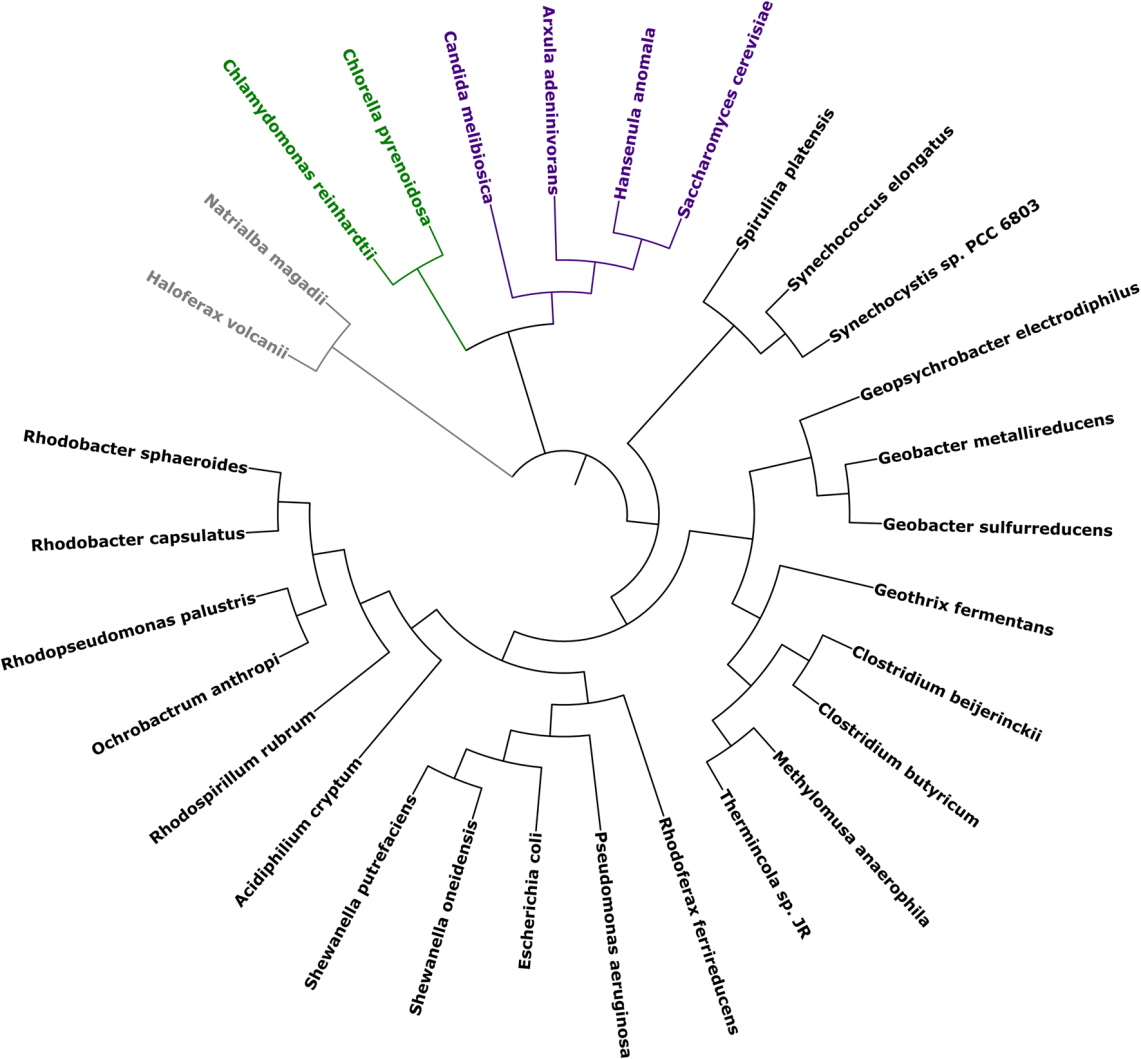
Phylogenetic analysis of typical electricigens used in MFCs based on 16S or 18S rRNA sequences. Phylogenetic analysis was conducted using the MEGA7 software [110]. Sequence alignment was performed by ClustralW. The phylogenetic tree was constructed using the Neighbor-Joining method. GenBank accession numbers for the 16S or 18S rRNA sequences are: *A. cryptum*, NR_025851; *A. adeninivorans*, AB018123; *C. melibiosica*, AB013503; *C. reinhardtii*, JN863299; *C. pyrenoidosa*, AB240151; *C. beijerinckii*, LC071789; *C. butyricum*, AB687551; *E. coli*, J01859; *G. metallireducens*, L07834; *G. sulfurreducens*, NR_075009; *G. electrodiphilus*, NR_042768; *G. fermentans*, U41563; *H. volcanii*, NR_028203; *H. anomala*, NG_062034; *M. anaerophila*, LC203074; *N. magadii*, NR_028243; *O. anthropic*, EU275247; *P. aeruginosa*, NR_026078; *R. capsulatus*, NR_043407; *R. sphaeroides*, NR_029215; *R. ferrireducens*, NR_074760; *R. palustris*, M59068; *R. rubrum*, NR_074249; *S. cerevisiae*, KU350743; *S. oneidensis*, NR_074798; *S. putrefaciens*, NR_044863; *S. platensis*, AB074508; *S. elongates*, NR_074309; *Synechocystis* sp. PCC 6803, AY224195; *Thermincola* sp. JR, GU815244

Generally, diverse of microorganisms have the potential to be used as the electricigens in MFCs. Figure 5 shows the phylogenetic analysis of typical electricigens in MFCs based on 16S or 18S rRNA sequences. These species could be divided into three distinct groups, the archaebacteria, the eubacteria and the eukaryotes. The eubacteria could be further classified into several subgroups according to their families and genera. There seems to be no direct correlation between the taxonomic status and their electricity-producing capability. Their electrochemical activities should be the result of convergent evolution.

## Mixed communities as electricigens in the anode

The activity of at least one electricigen is the requirement for MFCs to generate electricity. However, a diversity of electricigens can also contribute to current production and, in most cases, may be more efficient. The idea of using mixed communities has thus been proposed in the last decade. Pure cultures are useful to clarify the electron transfer mechanism at the microbiological level and further reduce the specific microbial strains in mixed cultures. However, pure cultured electricigens require relatively strict operating conditions and only selective substrates can be utilized while miscellaneous consortiums are more suitable for the use of complex substrates. MFCs with the best performance are always achieved by using mixed communities, such as wastewater or activated sludge, as the anodic biocatalyst.

### Wastewater species

Some species of the microorganisms in wastewater are electrochemical active. Therefore, wastewater could be directly employed as the inoculum for MFCs. Distillery wastewater contains organic substrates that can be easily degraded. It is a good source for electricity generation in MFCs. When diluted distillery wastewater was inoculated into the anode chamber, the peak power and current densities reached 168 mW/m^2^ and 580 mA/m^2^ [81]. Sugar beet processing wastewater was also used for electricity generation in a dual chamber MFC. Raw sugar beet processing wastewater was diluted to different concentrations and fed to the anode in batch-mode. A maximum power density of 14.9 mW/m^2^ was obtained [82]. Brewery wastewater was used as the substrates in an MFC with special chitosan copolymer proton exchange membrane. The maximum current and power densities of this MFC were 111.94 mA/m^2^ and 3022.39 mW/m^2^, respectively [83]. In a recent study, industrial wastewater from different sources including chocolate industry, gum industry and slaughterhouse industry were tested as the anodic catalyst. The MFC inoculated with slaughterhouse wastewater achieved the highest power density of 267 mW/m^2^ [84].

### Activated sludge

Activated sludge is a biological floc containing microbial communities as well as their dependent biodegradable organic compounds. It is also a good candidate as electricigens for MFCs [85]. Dentel et al. first demonstrated the direct generation of electricity from activated sludge. They applied anaerobic activated sludge as the anodic inoculum and observed a power voltage of 517 mV [86]. Activated sludge from biogas plants was used in MFCs to study the stability of the system and measure the power generated [87]. MFCs inoculated with three different types of activated sludges could simultaneously generate electricity and remove chemical oxygen demand. Heat and acid pretreatment of these sludges further improved their electricity production capacity [88]. Anaerobic sludge obtained from a wastewater treatment plant was used as mixed inoculums for a single-chamber MFC. The peak power density of 488 mW/m^2^ was achieved using ethanol as the substrates [89]. Enriched activated sludge was obtained by inoculation of fly ash leachate to different media. A maximum power density of 5.43 W/m^3^ was observed in the MFC by systematically optimizing anodic parameters [90].

The microbial populations in MFCs inoculated with wastewater or activated sludge vary greatly. To exploit the diversity of electricigens in the mixed communities, the 16S rRNA gene sequencing technique was employed to analyze the microbial community structure. Phylogenetic analysis showed that electricigens in these MFCs always consisted of the strains discussed above. For instance, Tkach et al. identified 59 strains in the mixed bacterial consortia of an MFC while *Geobacter* and *Pseudomonas* were the dominant genera [91]. The microbial communities are dynamically changed during the MFC operation processes and their dynamics can be also analyzed using similar strategies. The correlation between the power density and the changes of microbial species were determined by denaturing gradient gel electrophoresis of partial 16S rRNA genes [92].

### Defined co-cultures

The mixed communities in wastewater and activated sludge are very complex, making it difficult to elucidate how current is generated in such systems. Also, it is difficult to steer these mixed cultures to a stable performance. Therefore, scientists aimed to develop a defined co-culture consortium in which electricigens were rationally selected (Table 2). The co-culture system of *G. sulfurreducens* and *E. coli* was first established by Qu et al. The performance of this MFC was improved when compared with the pure culture of *G. sulfurreducens* due to the consumption of oxygen leaking into the reactor [93]. *P. aeruginosa* could synthesize electron mediators. Therefore, it is an excellent strain to construct co-culture systems. Defined co-cultures of *P. aeruginosa* and *Enterobacter aerogenes* were used as the anodic biocatalyst in an MFC. The fermentation parameters were studied to achieve higher electricity production and dissolved oxygen was identified as the key factor. After optimization of oxygen supply, 400% enhancement of current production was observed [94]. The defined co-culture system of *P. aeruginosa* and *Klebsiella variicola* was more efficient than either of these two strains alone, and the maximum current density was about 3 times higher [95]. Further optimization of the process parameters by response surface methodology led to a maximum power density of 12.88 W/m^3^ [96]. *Klebsiella pneumonia* was another strain that could secrete electron mediators. The MFC constructed by co-culture of *Klebsiella pneumonia* and *Lipomyces starkeyi* produced a peak power density of 12.87 W/m^3^ [97].

**Table 2:** Performance of MFCs constructed by defined co-cultured electricigens

Species 1	Species 2	Current density	Power density	References
*G. sulfurreducens*	*E. coli*	NR	918 mW/m^2^	[93]
*P. aeruginosa*	*E. aerogenes*	212 µA/cm^2^	NR	[94]
*P. aeruginosa*	*K. variicola*	NR	12.88 W/m^3^	[96]
*G. sulfurreducens*	*C. cellulolyticum*	NR	143 mW/m^2^	[99]
*S. oneidensis*	*S. cerevisiae*	369.4 mA/m^2^	123.4 mW/m^2^	[100]
*K. pneumonia*	*L. starkeyi*	NR	12.87 W/m^3^	[97]
*S. oneidensis*	*K. pneumonia*	10 mA/m^2^	2.15 mW/m^2^	[102]
*S. oneidensis*	*E. coli*	3.0 μA/cm^2^	NR	[103]

NR, not reported

Co-cultured microorganisms could expand the carbon source range in MFCs. Lignocellulosic biomass is the most abundant carbohydrate in nature [98]. Since no microorganisms can simultaneously degrade biomass and transfer electrons to the electrode, the utilization of cellulosic biomass as substrates for MFCs requires a co-cultured system. Ren et al. constructed an MFC inoculated with a defined co-culture of the cellulose-utilized strain *Clostridium cellulolyticum* and the electricigen *G. sulfurreducens*. This MFC produced peak power densities of 143 mW/m^2^ and 59.2 mW/m^2^ from two different kinds of cellulose, respectively [99]. *S. cerevisiae* and *S. oneidensis* co-culture consortium was rationally designed to establish a glucose-fed MFC. The co-culture conditions were systematically optimized to balance glucose catabolism and electron transfer. A maximum power density of 123.4 mW/m^2^ was finally achieved by this MFC [100]. At present, the combination of two species did not give high gains of efficiencies as wastewater or activated sludge species. However, defined co-culture MFCs provided a strategy to study the interaction between different microorganisms in miscellaneous consortia. The interaction of *G. sulfurreducens* and *E. coli* was investigated in their co-cultured MFCs. Metabonomics analysis showed that the consumption of both succinate and oxygen by *E. coli* would be helpful to increased current production by *G. sulfurreducens* [101].

## Conclusions and future perspectives

The MFC technology has been greatly advanced in the past few years. However, there are not yet any practical applications of MFCs due to the limitations of their power outputs. Electricigens are the fundamental issues in the MFC systems. The application of electricigens will be the focus of future research for MFCs. To improve the performance of an MFC, it is necessary to select and breed high-quality electricigens. Recently developed metabolic engineering and synthetic biology tools have been put forward to such an extent to modify current electricigens or create novel electricigens with excellent electrochemical activities [104]. Using genetic modification, the largely unexplored potential to improve power output and electron transfer to the electrode is almost limitless. The power densities are determined not only by the electricigens but also by the architecture, electrode spacing and electrolyte conductivity of MFCs [105]. The improvements in these aspects would also contribute to enhancing the power generation efficiency of an MFC.

Moreover, MFC should be integrated with other processes to make this technique economically feasible. First, MFC can be used for wastewater treatment. Application of MFC for wastewater treatment could be an attractive alternative to reduce the cost of existing systems. The power generated by MFCs would reduce the electricity required by the wastewater treatment bioreactors [106]. Second, MFC can be used to simultaneously produce valuable products (i.e., H_2_). The protons generated in the anode could migrate to the cathode to form H_2_. Compared with conventional biological H_2_ processes, MFC can potential produce 8–9 mol H_2_ per mol glucose instead of the theoretical yield of 4 mol H_2_/mol glucose [107]. Third, electricigens can sense both the presence and toxicity of chemicals. MFC-based biosensors are appropriate for real-time monitoring of environmental parameters [108]. At last, MFC for bioremediation is another promising application. MFCs have been proposed for the clean-up of various types of contamination, ranging from aromatic or substituted organic compounds to heavy metals [109]. During the bioremediation process, electricity is also generated and thus the cost is reduced. The combination of the MFC technology with other applications can make the dream of a possible large-scale launch of MFC come true.
